# Molecular and chemical characterization of mutant and nonmutant genotypes of saffron grown in Saudi Arabia

**DOI:** 10.1002/fsn3.875

**Published:** 2018-11-06

**Authors:** Mahmoud A. Sharaf‐Eldin, Pravej Alam, Shereen F. Elkholy

**Affiliations:** ^1^ Sara Alghonaim Research Chair (SRC), Biology Department College of Science and Humanities Prince Sattam bin Abdulaziz University Alkharj Saudi Arabia; ^2^ Non Traditional Spices Biotechnology Unit (NTSBU) Medicinal and Aromatic Plants Research Department National Research Centre (NRC) Cairo Egypt; ^3^ Department of Plant Molecular Biology Plant Transformation and Biopharmaceuticals Lab Agricultural Genetic Engineering Research Institute (AGERI) Agricultural Research Centre (ARC) Giza Egypt

**Keywords:** *Crocus sativus*, random amplified polymorphic DNA, saffron, safranal, stigmata

## Abstract

Saffron (*Crocus sativus* L.) is an important spice and medicinal plant that is cultivated in Asia, Europe, North Africa, and North America. Its morphological and biochemical parameters, such as the changes in the floral parts (six tepals, three stamens, three stigmata), biomass, and chlorophyll content, are primarily affected by environmental conditions. A polymerase chain reaction–rapid amplified polymorphic DNA (PCR‐RAPD) approach was used to analyze the extent of the polymorphisms between *C. sativus* genotypes grown in the Saudi climate. In this research study, the DNA fingerprints of the stigmata of *C. sativus* genotypes [K1 & K2 = *C. sativus* var. *cashmerianus*, C1 = *C. sativus* (nonmutant), T1 = mutant (T0‐2B), T2 = mutant (T1‐2B), T3 = mutant (T4‐2A)] were determined according to the floral parts, and a total of 10 decamer primers were used for PCR‐RAPD analysis. Only three pairs of arbitrary primers showed polymorphisms (33.3%–88.2%) in the total genomic DNA extracted from these genotypes. Jaccard's similarity index (JSI) ranged from 0.88 to 1.0. An unweighted pair group method with arithmetic mean (UPGMA) similarity and dendrogram matrix showed that two genotypes (T1‐2B and T4‐2A) were closely related to each other and to the strain CM‐cashmerianus, while the T0 of *C. sativus* genotype showed divergence.

## 1 INTRODUCTION

Saffron is the dehydrated stigmata of *Crocus sativus* and is considered the most expensive spice in the world (Busconi et al., 2015; Sharaf‐Eldin et al., 2008; Sharaf‐Eldin et al., 2015). It is an autumn‐flowering perennial plant that is mainly used in food as a colorant spice and fragrance, and more than 85% of worldwide saffron production occurs in Iran (FAO, 2012). Saffron is characterized by its bitter taste due to the presence of safranal and the crocin complex (Negbi, 1999; Sharaf‐Eldin et al., 2015). These traits make saffron a highly sought‐after ingredient for many foods worldwide.

The taxonomy of *Crocus* is very convoluted due to its sterility, triploidy (2n = 3x = 24), and the heterogeneity of both its morphological traits and cytological records (Rubio‐Moraga, Castillo‐López, Gómez‐Gómez, & Ahrazem, 2009). In many crop species, while genetic relationships based on molecular markers have been consistent with expectations from pedigrees and breeding behavior, techniques for estimating genetic variability, such as analyzing physiochemical markers, do not yield enough polymorphisms to detect genetic differences between genotypes (Busconi et al., 2015; Goodman & Stuber, 1980; Smith, Goodman, & Stuber, 1984). Taxonomically, therefore, morphological traits such as growth habit, floral morphology, leaves, and fruits have been used to classify plants. The use of molecular techniques, therefore, helps a taxonomist not only to identify genotypes but also to assess and exploit genetic variability via molecular markers (Whitkus, Doebley, & Wendel, 1994).

Saudi Arabia is one of the countries with the highest levels of consumption of saffron spice. In the year 2009, the price for one kg of saffron spice in the local market reached 18,000 SR (~US$ 5,000). The development of domestic saffron production in Saudi Arabia is therefore important. In this context, Sharaf‐Eldin, Fernandez, Al‐Khedhairi, and Elsayed (2013) reported, for the first time, the cultivation of saffron in the Kingdom of Saudi Arabia (KSA), in particular within Alkharj Governorate, and recommended the cultivation of saffron corms in the first half of September, with the expectation of harvesting in the fourth week of November.

Polymerase chain reaction–random amplified polymorphic DNA (PCR‐RAPD) is an important tool for identifying molecular markers and differentiates plant genomes on the basis of genomic DNA banding (Tivang, Skroch, Nienhuis, & De Vos, 1996; Williams, Kubelik, Livak, Rafalski, & Tingey, 1990). The polymorphisms and variability of genomic DNA can be seen after performing electrophoresis on the DNA bands that result from the primer binding sites. Significant variations in plant genome size occur in different species and differentiate the molecular characteristics of plants, allowing phylogenetic relationships to be established, as recognized by Goodspeed (1954). Molecular markers derived from PCR‐based RAPD, as described by Williams et al. (1990), are relatively easy to generate and are inexpensive. Compared with other DNA‐based marker methods, RAPD is a simple and inexpensive molecular marker technique; therefore, it has been used to differentiate close variants of plant species such as artichoke (Tivang et al., 1996), *Echinacea* (Nieri et al., 2003), *Astragali radix* (Na et al., 2004), turmeric (Sasikumar, Syamkumar, Remya, & John Zachariah, 2004), *Dendrobium officinale* (Ding et al., 2005), *Dendrobium* species (Zhang, But, Wang, & Shaw, 2005), watermelon (Levi et al., 2017), *Typhonium* species (Acharya, Mukherjee, Panda, & Das, 2005), and *Tinospora cordifolia* (Rout, 2006). We conducted the experiment reported in this paper to identify the genotypic variations in growth traits and constituent contents in relation to genotypic differences, mutant (five‐stigmata) and nonmutant (three‐stigmata) saffron.

## 2 MATERIALS AND METHODS

### 2.1 Plant material

**Figure 1:**
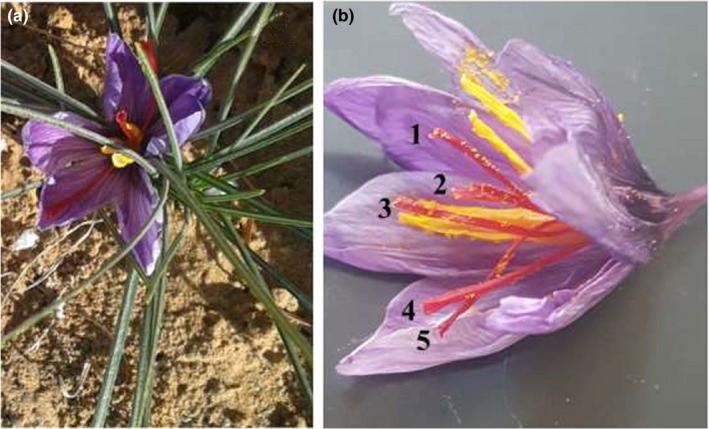
*Crocus sativus* plant showing three and five stigmata A = normal or nonmutant flower (three stigmata); B = mutant flower (five stigmata)

Corms (each 10–12 g) of *C. sativus* L., and *C. sativus* var. cashmerianus (Iridaceae) from Dix Export b.v., the Netherlands, were cultivated during the September 2013 and 2014 growing seasons at the experimental farm station managed by the Sara bint Rached bin Ghonaim Research Chair (SRC) for Cultivating nonTraditional Medicinal and Aromatic Plants, College of Science and Humanities, Prince Sattam bin Abdulaziz University, Alkharj (24°04′N, 47°08′E), Saudi Arabia (Figure 1). This is a semiarid region, with an average annual rainfall of 15–25 mm. The main weather information for Alkharj, KSA, concerning temperature (T) is given in Table 1. Physical and chemical analyses of the field experimental soil (Table 2) were carried out before planting followed the method of Chapman and Pratt (1978). Saffron flowers with five or three stigmata were harvested during November and December of both growing seasons (2013 and 2014).

**Table 1:** The mean temperature (°C) in Alkharj city throughout the experimental period

	September	October	November	December	January	February	March	April	May	June	July	August
Year 2013												
Max T (°C^a^	42	34	30	22	24	27	31	33	38	40	42	45
Min T (°C^a^	26	21	9	5	9	12	15	20	26	32	31	31
Year 2014												
Max T (°C)	38	35	28	35	23	27	30	37	42	43	41	41
Min T (°C)	28	19	13	11	6	11	12	19	31	32	35	28

Mean temperature.

**Table 2:** Properties of the soil used for growing saffron

Clay (%)	Silt (%)	Sand (%)	Corg. (%)	OM1 (%)	pH	EC (dSm‐1)	N2 (ppm)	P3 (ppm)	K3 (ppm)
17.2	8.2	74.8	0.36	0.62	7.76	1.47	16.6	14.2	153.29

1: organic matter, 2: total, 3: available.

### 2.2 Experimental design

We set up the experiment in a randomized complete block design with three replicates in each growing season. Plant population is based on the spacing between plants within row (intrarow = 20 cm) as well as the spacing between two adjacent rows (inter‐row = 1 m) had been designed. Each plot was 2 m long and had two rows 1 m apart with 20 corms in each. The corms were planted at a depth of 10 cm, and we took care to provide all necessary agricultural support [irrigation (drip method), fertilizer, and weed control] to ensure unstressed conditions (Sharaf‐Eldin, Fernandez, Al‐Khedhairi, & Elsayed, 2013).

### 2.3 Flower samples

About 75 days after the corms were planted, we harvested the saffron flowers. This was done by hand early every morning before the tepals started to open. The crop cycle of saffron was estimated as 6 months. We harvested the first flower at the end of November and the last three weeks after that. The biomass data were assessed based on the floral dry weight, including the stigma, stamen, and tepal.

### 2.4 Genomic DNA extraction

We isolated genomic DNA from *C. sativus* L. genotypes in our laboratories using a modified version of Doyle and Doyle's protocol (1990). The DNA was purified from contaminants such as RNA, proteins, phenols, terpenes, and other secondary metabolites, as well as the colored compounds present in the *C. sativus* leaves. The purity of the DNA was checked on a 1.0% agarose gel and resolved to appear as fine bands. We then treated the extracted DNA sample in TE with 1 μl RNase (10 mg/ml stock) for 10 min at 37°C, and then added an equal volume of the mixture of chloroform: isoamyl alcohol (24:1). We centrifuged the mixture at 11,180 *g* for 10 min at 4°C, before extracting the aqueous phase and adding 0.6 volume of isopropanol. We then froze the mixture at −20°C for 10 min before centrifuging it once again at 10,000 rpm for 5 min at 4°C and carefully decanting the supernatant. We washed the resulting pellet twice with 70% ethanol and dried it at 37°C for 10 min. Finally, the DNA pellet was dissolved in 50 μl of 1× TE buffer. The absorbance of the DNA solution was taken at 260 nm using a spectrophotometer (Specgen, Darmstadt, Germany) in order to determine the concentration and purity of the DNA samples. For double‐stranded DNA, an absorbance of 1.75 at 260 nm corresponds to a concentration of 30 μg/ml. The DNA sample was then diluted, and absorbance was observed at 260 nm against a blank reference (control without DNA and with only a TE buffer).

### 2.5 Generation of randomly amplified polymorphic DNA (RAPD) markers

We extracted DNA from leaf samples of *C. sativus* genotypes (CM‐cashmerianus, T0, T1‐2B, T4‐2A) and subjected these DNA samples to RAPD analysis. To this end, we undertook PCR amplification with 10 sets of random primers (Table 3). The PCRs were conducted in 100‐μl Eppendorf tubes in a final volume of 25 μl, which contained the template (15 ng/μl), dNTP mix (2.5 mM each), Taq DNA polymerase, reaction buffer (10×), and 50 ng of random primers, adjusting the final volume to 25 ml with distilled water. The PCR amplification was conducted by using the Applied Biosystems, USA, using the following conditions: initial denaturation at 94°C for 5 min, followed by 40 cycles of denaturation at 94°C for 1 min, annealing at 35°C for 1 min, and extension at 72°C for 2 min; a final extension at 72°C for 5 min; and holding at 4°C. The PCR products were electrophoresed on 1.2% agarose gels and photographed using a gel documentation system (Bio‐Rad, Gladesville, Australia).

**Table 3:** Arbitrary random primers used in random amplified polymorphic DNA analyses

Name	Primer sequence
GCA01	CAGGCCCTTC
GCA02	TGCCGAGCTG
GCA03	AGTCAGCCAC
GCA04	AATCGGGCTG
GCA05	AGGGGTCTTG
GCA06	GGTCCCTGAC
GCA07	GAAACGGGTG
GCA08	GTGACGTAGG
GCA09	GGGTAACGCC
GCA10	GTGATCGCAG
GCA11	CAATCGCCGT
GCA12	TCGGCGATAG
GCA13	CAGCACCCAC
GCA14	TCTGTGCTGG
GCA15	TTCCGAACCC
GCA16	AGCCAGCGAA
GCA17	GACCGCTTGT
GCA18	AGGTGACCGT
GCA19	GTTGCGATCC
GCA20	CAAACGTCGG

### 2.6 Phylogenetic analyses using RAPD fingerprints

We used the ten random primer sets to amplify all possible and reproducible monomorphic and polymorphic bands for the initial screening against the *C. sativus* genotypes (CM‐cashmerianus, T0, T1‐2B, T4‐2A) so as to identify RAPD markers (Table 3). Each sample was run in triplicate during PCR to verify its reproducibility. The RAPD primers were classified based on the manufacturer's primer code and corresponded to the 10‐mer oligo used in this study followed by a four‐digit number that indicated the size of the product in base pairs. The phenotype of each RAPD marker was scored as 1 when bands were present and 0 when no bands were observed. We performed a genotype cluster analysis to generate a dendrogram based on Jaccard's similarity coefficients earlier used by Sharaf‐Eldin et al. (2015) and unweighted pair group mean analyses (UPGMA) with NTSYS‐PC ver. 2.0 to develop the phylogenetic tree (Rohlf, 2001).

### 2.7 Estimation of chlorophyll content

Leaf chlorophyll content is a recognized indicator of many concerns, such as plant photosynthesis activity, plant nutritional state, presence of mutations, and stresses. We therefore determined the total chlorophyll content of the leaves of the *C. sativus* genotypes (CM‐cashmerianus, T0, T1‐2B, T4‐2A) using the method of Hiscox and Israelstam (1979). Specifically, we used dimethyl sulfoxide at 65°C for 2.5 hr to isolate the chlorophyll from 100 mg samples of *C. sativus* leaves that were taken during early December. We then measured the absorbance of the reaction mixture at 645 and 660 nm in a UV‐Vis spectrophotometer (Spectroscan 80 DV, USA), and after it was recorded, the total chlorophyll content was calculated (Arnon, 1949).

### 2.8 Extraction and HPLC analyses of the crocin and safranal content of saffron genotypes

Fifteen milligrams of saffron stigmata dried at 60°C (CM‐cashmerianus, T0, T1‐2B, T4‐2A) was used to extract the metabolites. We suspended the samples in 10 ml of methanol–water (50:50, v/v) and mixed them using a magnetic stirrer for 24 hr at room temperature in the dark according to Alam, Elkholy, Hosokawa, Mahfouz, and Sharaf‐Eldin (2016). After extracting the crocin (440 nm) and safranal according to Alam et al. (2016), we filtered the samples through a 0.25‐μm filter membrane (Millipore, Bedford, MA, USA) and stored at 4°C for HPLC analysis.

Rapid HPLC analysis was performed on a multisolvent Agilent 1260 Infinity Quaternary LC system. The Agilent Open LAB ChemStation version C.01.05 (Agilent, Lexington, MA, USA) was used for data acquisition and chromatogram processing on the basis of the area and retention time. The analyses were conducted in triplicate for each sample, and the concentrations of crocin and safranal are expressed in milligrams per gram (mg/g) of saffron stigmata. All the chemicals, including standard crocin, safranal, methanol, and ethanol were purchased from Sigma (St. Louis, MO, USA).

### 2.9 Statistical analyses

The treatments in these experiments were arranged in a randomized complete block design. Each treatment included three sets of replicate data for each season and was statistically analyzed applying the ANOVA test (MS DOS/Costat Exe Program) according to Gomez and Gomez (1984). The least significant difference (at a level of 5%) was used to compare between different means, according to Snedecor and Cochran (1982).

## 3 RESULTS

### 3.1 DNA isolation, purification, and quantification

Genomic DNA was isolated from each genotype of *C. sativus* (CM‐cashmerianus, T0, T1‐2B, T4‐2A) according to Doyle and Doyle (1990) with our own modifications; as the quality and quantity of the isolated DNA were determined by using optical density (OD) at 260/280 nm on a spectrophotometer. The results showed that the yield of genomic DNA among the *C. sativus* genotypes varied from 112 to 167 ng.

### 3.2 Generation of RAPD fingerprints from the saffron genotypes

**Figure 2:**
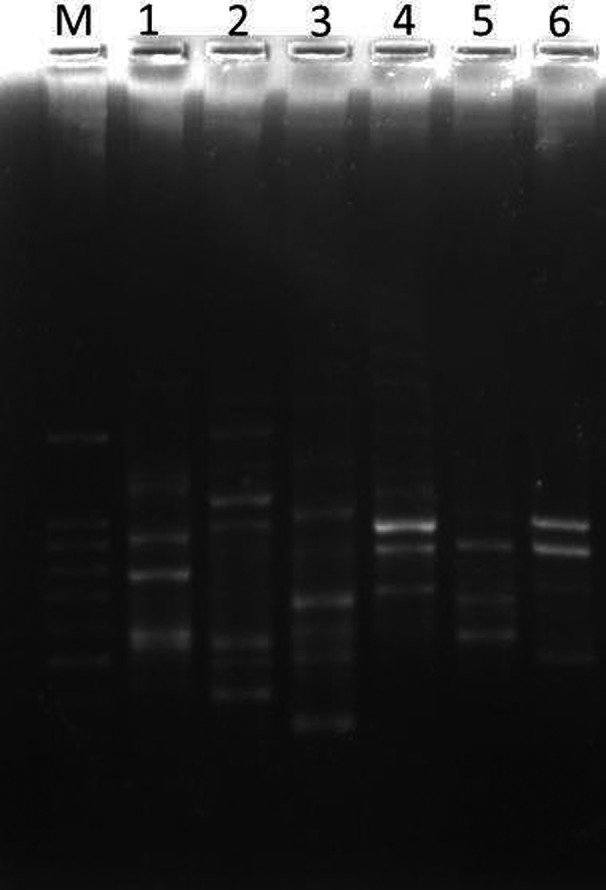
Random amplified polymorphic DNA profiling of mutant and nonmutant genotypes of *Crocus sativus*, M: 100 bp DNA ladder; 1: CM1 (K1); 2: CM2 (K2); 3: T0 (C1 = nonmutant); 4: mutant (T1 = T0‐2B); 5: mutant (T2 = T1‐2B); 6: mutant (T3 = T4‐2A)

Different RAPD fingerprints were obtained from all the saffron genotypes. Twenty random GCC primers were used for the RAPD analysis. The RAPD profile showed monomorphisms (similar bands) but also polymorphisms that were unique to the selected random GCC primer. The sizes of the bands varied in the range of 100–1,000 bp for both the monomorphic and polymorphic forms (Table 4, Figure 2). The amplification pattern was more pronounced with GCA‐01 (CAGGCCCTTC), while the other random primers were unable to amplify uniformly. GCC‐01, however, amplified the genomic DNA of all saffron genotypes and generated unique fragments.

**Table 4:** Analyses of polymorphisms on the basis of random amplified polymorphic DNA profiling

Genotype name	No. of monomorphic bands	No. of polymorphic bands	Total no. of bands
*Crocus sativus* var. *cashmerianus* (CM1 = K1)	0	4	4
*C. sativus* var. *cashmerianus* (CM2 = K2)	1	4	5
*C. sativus* [control^a^] (T0 = C1)	2	7	9
T0‐2B (T1)	1	6	7
T1‐2B (T2)	1	6	7
T4‐2A (T3)	1	3	4

a Control genotypes bearing three stigmata.

### 3.3 Analysis of the genetic similarity between the saffron genotypes

On the basis of the RAPD fingerprint polymorphisms between the genotypes of saffron bearing five stigmata, a similarity matrix was obtained after multivariate analysis using the “Nei and Li” coefficient (Nei & Li, 1979); this matrix is presented in Table 5.

**Table 5:** Genetic similarity between genotypes

	K1	K2	C1	T1	T2	T3
K1	0	0.471	0.882	0.667	0.667	0.333
K2		0	0.745	0.471	0.471	0.333
C1			0	0.577	0.577	0.816
T1				0	0.000	0.577
T2					0	0.577
T3						0

K1 & K2 (CM1 & CM2) = *Crocus sativus* var. *cashmerianus*, C1 (T0) = *Crocus sativus* (nonmutant), T1 = mutant (T0‐2B), T2 = mutant (T1‐2B), T3 = mutant (T4‐2A).

The genetic similarity matrix coefficients indicate that *C. sativus* var. *cashmerianus* (CM1 & CM2 = K1 & K2) shared approximately 88.2%, 66.7%, and 33.3% similarity with (C1 = T0 nonmutant), [(T1 mutant = T0‐2B) & (T2 mutant = T1‐2B)] and (T3 mutant = T4‐2A) (Table 5), we enforced to use different nomenclatures for each genotype according to the compatibility of each software (CM1 = K1, C1 = T0, etc). The phylogenetic tree revealed the distances among all *Crocus* genotypes, as shown by the numerical taxonomy.

**Figure 3:**
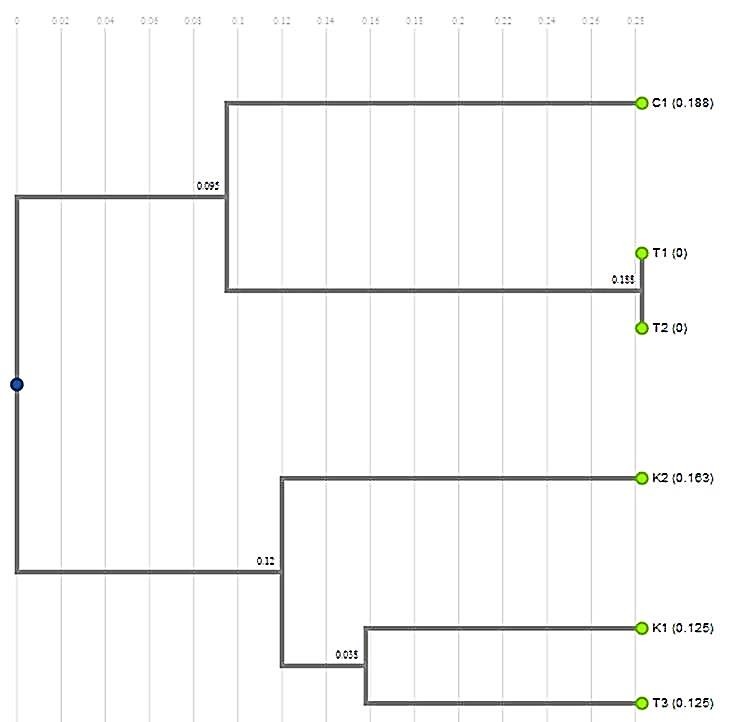
Phylogenetic analysis of mutant and nonmutant genotypes of *Crocus sativus*. K1 & K2 (CM1 & CM2) = *C. sativus* var. *cashmerianus*, C1 = *C. sativus* (T0 = nonmutant), T1 = mutant (T0‐2B), T2 = mutant (T1‐2B), T3 = mutant (T4‐2A)

The dendrogram of the *Crocus* genotypes clearly indicated that the PCR‐RAPD correlated with the similarities and distances between the *C. sativus* genotypes, from which one could to a large extent predict the origin of the species (Figure 3). PCR‐RAPD also showed the mutational pattern among the genotypes.

### 3.4 Biomass

The dry biomass of the stigmata, stamens, and tepals was studied in genotypes of *C. sativus*. The dry biomass of the stigmata of T0‐2B (10 mg/flower), CM (6.8 mg per flower), and T1‐2B (5.8 mg per flower) was higher than that of the T0 stigmata (5.2 mg per flower), while the dry biomass of the tepals did not differ in any of the mutant flowers (10 mg per flower) with respect to the control T0 (Table 6).

**Table 6:** Analysis of the biomass (dry weight basis [mg per flower]) of the floral parts of *Crocus sativus* genotypes

Genotypes	Stigma	Stamen	Tepal
*C. sativus* var. *cashmerianus* (CM = K)	6.8	5.7	10.0
*C. sativus* (control) (T0 = C1)	5.2	7.0	10.0
T0‐2B (T1)	10.0	10.0	10.0
T1‐2B (T2)	5.8	10.0	10.0
T4‐2A (T3)	4.2	10.0	10.0
LSD_(0.05)_	0.068	0.080	0.089

Note

LSD: least significant difference.

### 3.5 Chlorophyll content

The chlorophyll content in CM (17.45 mg/g fw), T0‐2B (21.90 mg/g fw), T1‐2B (18.56 mg/g fw), and T4‐2A (19.25 mg/g fw), as well as the nonmutant genotype T0 (with three stigmata) (21.90 mg/g fw), was recorded and is shown in Table 7.

**Table 7:** Total chlorophyll content (mg/g on a fresh weight basis) of the leaves of *Crocus sativus* genotypes

Treatment	Total chlorophyll (mg/g)
*C. sativus* var. *cashmerianus* (CM = K)	17.45
*C. sativus* (control) (T0 = C1)	21.90
T0‐2B (T1)	21.90
T1‐2B (T2)	18.56
T4‐2A (T3)	19.25
LSD_(0.05)_	0.709

Note

LSD: least significant difference.

### 3.6 Analysis of crocin and safranal

The crocin and safranal concentrations were evaluated in the saffron genotypes. The crocin content in genotypes with five stigmata, CM (27.36 mg/g), T0‐2B (14.85 mg/g), T1‐2B (13.05 mg/g), and T4‐2A (12.03 mg/g), was greater than that in the T0 (9.9 mg/g), while the safranal content of CM (1.4 mg/g), T0‐2B (1.0 mg/g), T1‐2B (1.0 mg/g), and T4‐2A (1.6 mg/g) was similar to that of the T0 (1.4 mg/g) genotypes, as shown in Table 8.

**Table 8:** Analysis of safranal and crocin in *Crocus sativus* genotypes

Genotypes	Safranal	Crocin
*C. sativus* var. *cashmerianus* (CM = K)	1.4	27.36
*C. sativus* (control) (T0 = C1)	1.4	9.9
T0‐2B (T1)	1.0	14.85
T1‐2B (T2)	1.0	13.05
T4‐2A (T3)	1.6	12.03
LSD_(0.05)_	0.447	0.345

Note

LSD: least significant difference.

## 4 DISCUSSION

Saffron (*C. sativus* L.) is one of the most valuable and expensive spices; it has high medicinal value and is used in the treatment of many diseases. The production of saffron is a very low worldwide because of its growth characteristics and high demand for labor (Sharaf‐Eldin et al., 2008).

*Crocus sativus* is a triploid (2n = 3x = 24) sterile plant; it fails to produce viable seeds and is totally dependent on human support (Rubio‐Moraga et al., 2009). In the recent past, it has been produced by breeders, which provides a better platform for maintaining its genetic balance. The quality of saffron mainly depends upon stigma processing and species origin (Sharaf‐Eldin et al., 2008). Caiola, Somma, and Lauretti (2000) reported that the phenological evaluation of *C. sativus* flowers obtained from corms from various regions of the world showed some variation in the color of the flowers as well as their fragrance and tepal lobes, but not in the pollen size (Caiola et al., 2000). Improvements in DNA biology have provided new information for taxonomic analysis among accessions (Caiola, Caputo, & Zanier, 2004), and some studies have demonstrated that the relationship between molecular and biochemical characterization is sufficiently robust to classify and clarify the systematics and phylogeny of plants (Frello & Heslop‐Harrison, 2000; Alavi‐Kia, Mohammadi, Aharizad, & Moghaddam, 2008; Rubio‐Moraga et al., 2009; Seberg & Petersen, 2009). PCR‐based analyses are in demand because they are simple, require only small quantities of genomic DNA, and are not very time‐consuming (Srivastava et al., 2012).

The advantages of the PCR‐RAPD technique include its rapidity; the low concentration of genomic DNA, dNTPs, and primers needed; and the ability to obtain genetic information without the use of radioisotopes (Srivastava et al., 2012; Williams et al., 1990). The reproducibility of PCR‐RAPD is affected by the quality of the DNA, the concentration of genomic DNA and the primers, and the source of the DNA polymerase (Ellsworth, Rittenhouse, & Honeycutt, 1993). The random primers used in RAPD are decamers (Williams et al., 1990) and are mostly designed based on microsatellite/minisatellite regions, which contain highly repetitive sequences. The PCR conditions used in this study for the RAPD analysis of the saffron genomic DNA were 94°C for denaturation, 35°C for annealing, and 72°C for elongation, repeated for 40 cycles. These conditions yielded the most bands. This result could be due to the low annealing temperature (35°C), which could allow maximum primer–DNA annealing and the maximum number of amplicons. These types of parameters have also been used by other investigators in different plant species (Busconi, Sebastiani, & Fogher, 2006; Miller & Bayer, 2001; Srivastava et al., 2012).

The morphological traits (e.g., tepal, stamen, and stigma) of *C. sativus* might be affected by the environment, and thus, the use of morphological traits for taxonomy or classification could result in incongruities. The effectiveness of a molecular marker technique depends on the quantity of polymorphisms that can be detected among the set of accessions under investigation (Singh, Srivastava, Srivastava, & Srivastava, 2011). The knowledge of genetic variations and relationships among the accessions or genotypes is an important basis for classification, germplasm resource utilization, and breeding for future use. Phylogenetic analysis (Figure 3) showed that the *C. sativus* genotypes obtained from the semiarid zone of Saudi Arabia were broadly divided into three main species. T0‐2B and T1‐2B were quite divergent, however, and did not fall into any of the major clusters. A notable genetic resemblance was observed in some of the genotypes analyzed, as shown by the high value of the similarity index. Based on the similarity index using simple matching coefficients, the similarity values between all *C. sativus* genotypes ranged from 33.3 to 88.2% of RAPD, as shown in Table 5. This finding could be due to the effect of the climatic conditions on the different saffron genotypes. This study provides strong proof that RAPD polymorphisms can be used as an important tool to reveal phylogenetic relationships among species and genotypes. Our results are also supported by observations from several investigators (Heikal, Abdel‐Razzak, & Hafez, 2008; Li, Fatokun, Ubi, Singh, & Scoles, 2001).

Similarly, the chlorophyll and biomass were also affected in these genotypes. The chlorophyll content of the T0 genotype (with three stigmata) (21.90 mg/g fresh weight basis) was higher than that of CM (17.45 mg/g), T1‐2B (18.56 mg/g) and T4‐2A (19.25 mg/g); the biomass of the stigma, stamen, and tepal varied among the *Crocus* genotypes CM (6.8 mg per flower), T1‐2B (5.8 mg per flower), T4‐2A (4.2 mg per flower) relative to that of the genotypes T0 (5.2 mg per flower); and the biomass of the tepals did not differ between any of the genotype flowers (10 mg per flower) and the control T0, as shown in Tables 6 and 7.

The crocin and safranal contents also varied, and the crocin content analysis of those genotypes with five stigmata yielded CM (27.36 mg/g), T1‐2B (13.05 mg/g), and T4‐2A (12.03 mg/g), higher than that of the T0 (9.9 mg/g). The safranal content analysis showed that CM (1.4 mg/g), T1‐2B (1.0 mg/g), and T2‐2A (1.6 mg/g) were similar to the T0 (1.4 mg/g) (Table 8). These differences are due to environmental conditions, which cause changes in the biochemical profile, genome methylation, or gene expression. These results were also supported by other investigators (Fernàndez‐Martínez, Zacchini, Elena, Fernández‐Marín, & Fleck, 2013; Smith, Burritt, & Bannister, 2000).

## 5 CONCLUSION

The main goals of this study were to explore the genetic relationships among genotypes or accessions by gaining a better understanding of the genetic differences among them. Furthermore, reproducible DNA markers, such as inter simple sequence repeats (ISSRs) and sequence characterized amplified regions (SCARs), can also support relationships among accessions or genotypes or species of the plants. Thus, PCR‐RAPD could be helpful in the identification of commercial saffron lines and could be a useful tool to supplement uniformity, distinctness, and stability analyses for saffron genotypes to maintain their original identity and protect the crop in the future.

## CONFLICT OF INTEREST

The authors declare no conflict of interests.

## ETHICAL STATEMENT

This article does not contain any studies with human participants or animals performed by any of the authors.
